# A novel data storage logic in the cloud

**DOI:** 10.12688/f1000research.7727.2

**Published:** 2016-03-29

**Authors:** Bence Mátyás, Máté Szarka, Gábor Járvás, Gábor Kusper, István Argay, Alice Fialowski

**Affiliations:** 1 Kerpely Kalman Doctoral School, Universidad Politécnica Salesiana, Rumichaca y Morán Valverde s/n, Debrecen, Ecuador; 2 Medical and Health Sciences Center, Research Centre for Molecular Medicine, University of Debrecen, Debrecen, Hungary; 3 Vitrolink Biotechnological Researching, Development,Servicing and Trading Limited Liability Company, Debrecen, Hungary; 4 Hungarian Academy of Sciences MTA-PE, Translational Glycomics Group, University of Pannonia, Veszprem, Hungary; 5 Computer Science Department, Eszterhazy Karoly University, Eger, Hungary; 6 Department of Obstetric and Gynecology, UD MSHC, University of Debrecen Medical Center, Debrecen, Hungary; 7 IRCAD France Laporoscopic Training center, Strasbourg, France; 8 Institute of Mathematics and Informatics, University of Pécs, Pécs, Hungary

**Keywords:** Joker Tao, NoSQL, Cloud, Database, Life science, Physical data table, Virtual data table, RDBMS

## Abstract

Databases which store and manage long-term scientific information related to life science are used to store huge amount of quantitative attributes. Introduction of a new entity attribute requires modification of the existing data tables and the programs that use these data tables. The solution is increasing the virtual data tables while the number of screens remains the same. The main objective of the present study was to introduce a logic called Joker Tao (JT) which provides universal data storage for cloud-based databases. It means all types of input data can be interpreted as an entity and attribute at the same time, in the same data table.

## Introduction

Databases which store and manage long-term scientific information related to life science are used to store huge amount of quantitative attributes. This is specially true for medical databases ^1, 2^. One major downside of these data is that information on multiple occurrences of an illness in the same individual cannot be connected ^1, 3, 4^. Modern database management systems fall into two broad classes: Relational Database Management System (RDBMS) and Not Only Structured Query Language (NoSQL) ^5, 6^. The primary goal of this paper is to introduce a novel data storage logic which provides an opportunity to store and manage each input data in one (physical) data table while the data storage concept is structured. JT can be defined as a NoSQL engine on an SQL platform that can serve data from different data storage concepts without several conversions.

## Methods

The technical environment is Oracle Application Express (Apex) 5.0 cloud-based technology. Workstation: OS (which is indifferent) + internet browser (Chrome). The Joker Tao logic ( www.jokertao.com) can be applied in any RDBMS system (e.g. www.taodb.hu). Specification of the physical data table structure was determined with - *ID* (num) as the identifier of the entity, which identifies the entity between the data tables (not only in the given data table); - *ATTRIBUTE* (num) is the identifier of the attribute; - *SEQUENCE* (num) which is used in the case of a vector attribute; and - *VALUE* (VARCHAR2) which is used for storing values of the attributes.

Data storage structure in JT logic based databases For the unique identification more attributes (columns) are applied in rational databases. In practice, attributes within a type are defined in same data table. Introducing new entity attributes requires the modification of the existing data tables and applications that use these data tables. In JT logic based databases, records with the same ID values identify one entity called virtual record. Virtual records with the same value of the “belonging to the virtual data table” attribute form a data table called virtual data table. Click here for additional data file. Copyright: © 2016 Mátyás B et al. 2016 Data associated with the article are available under the terms of the Creative Commons Zero "No rights reserved" data waiver (CC0 1.0 Public domain dedication).

**Figure 1.**
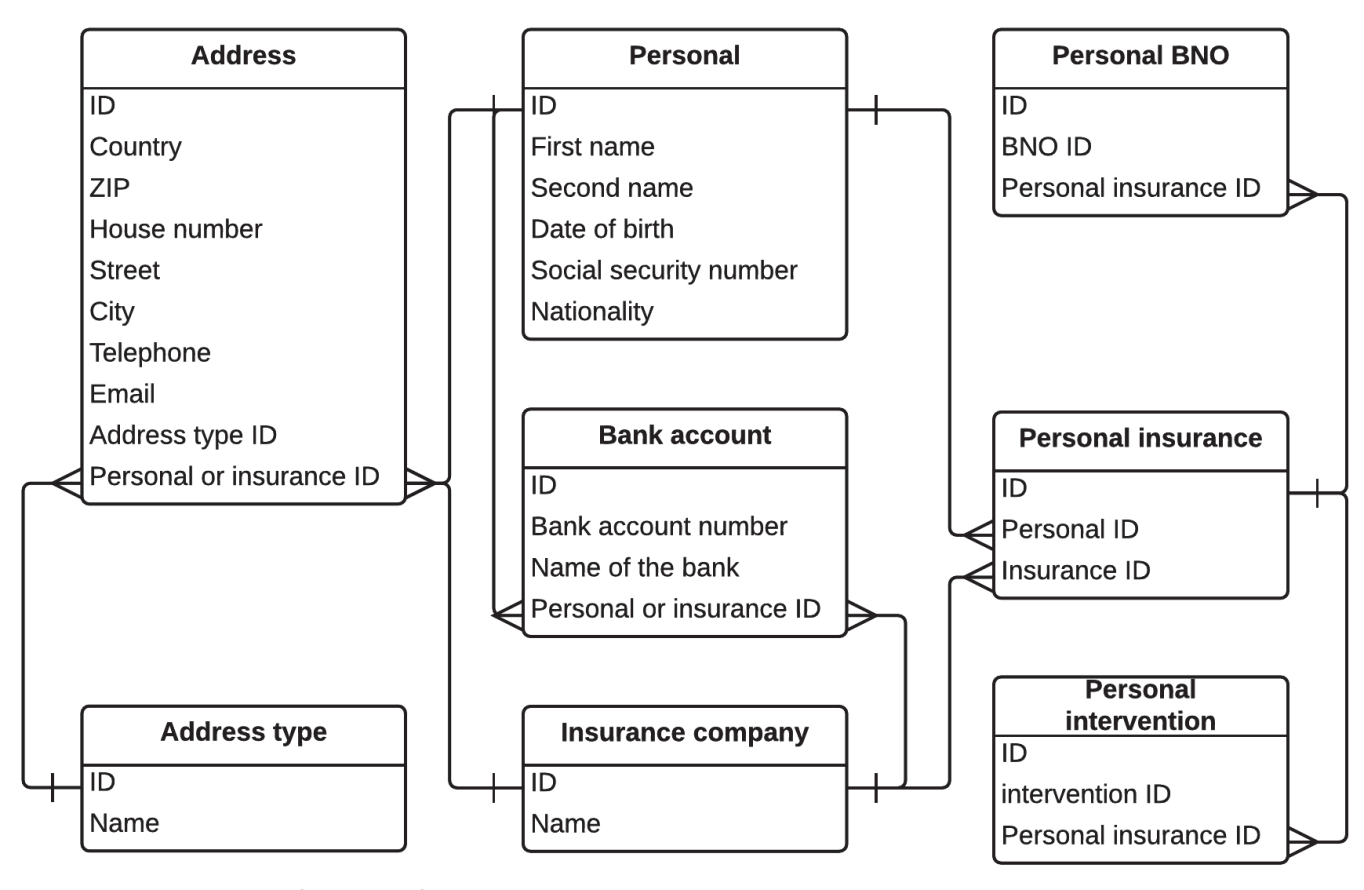
Example for a traditional (relational) data storage structure.

The codes which are stored in the *Attribute* column are also defined, sooner or later, in the *ID* column. At that time the attribute becomes an entity. In every case, the subjectivity determines the depth of entity-attribute definition in the physical data table. Firstly, we demonstrate a simplified relational database model ( Figure 1).

**Figure 2.**
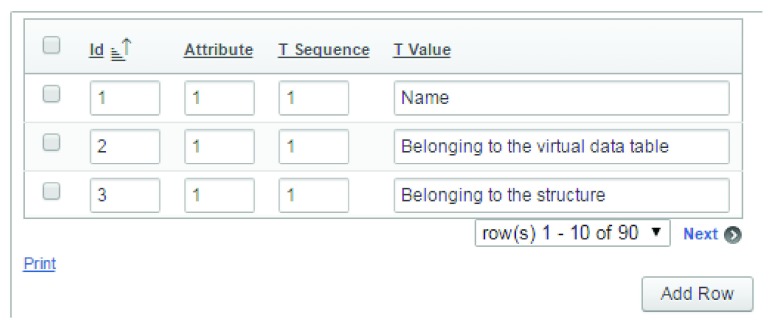
Basic attributes storage.

Following this, the presented data tables have been modified step by step. At the end of these steps, each data from the presented database will be stored in one physical data table using JT logic. The first step is the technical data storage. In Figure 2, basic relationships will be stored which help to describe the names of attributes (columns), type of relationships (belonging to the structure) and virtual data tables (belonging to the virtual data table).

**Figure 3.**
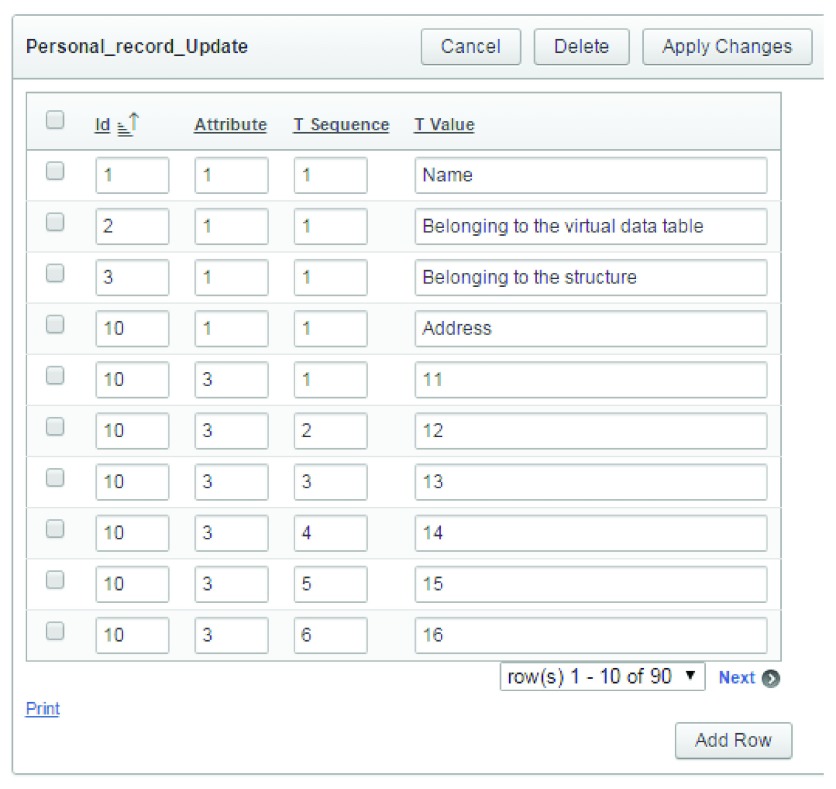
Entity storage.

In the second step, the records witch form virtual records are displayed ( Figure 3). The physical records with the same ID values mean a virtual record (entity) in the JT logic based databases. These identifiers can be any natural number that has not already been used in the ID column.

**Figure 4.**
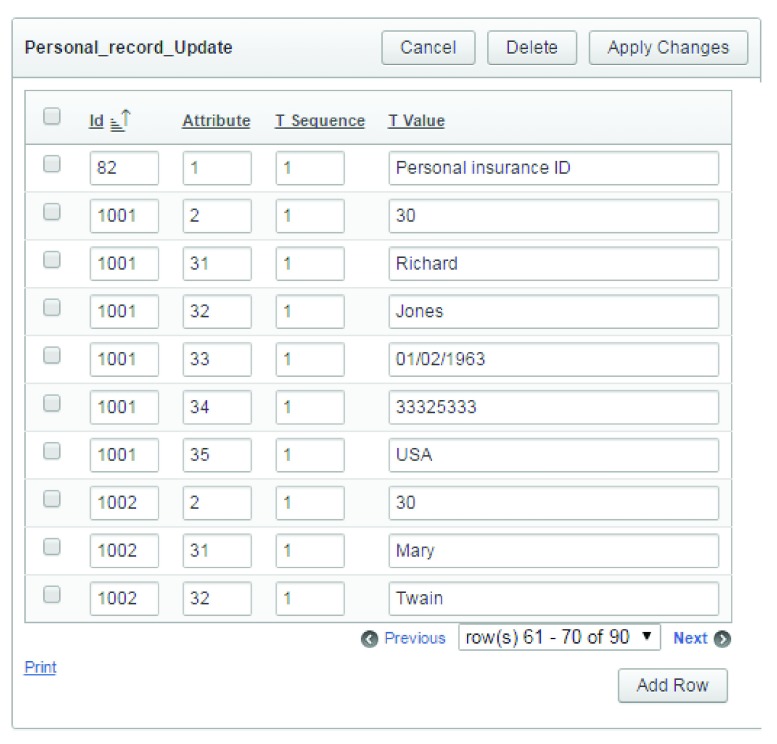
Attribute storage.

In the third step, records witch form new attributes are also displayed ( Figure 4). The values of these identifiers can be any natural number that has not already been used in the *Attribute* column.

Each attributes are identified in the Attribute column. In this case the following contexts can be read out related to the entity identified with 1001 ID value: -The value of the "belonging to the virtual data table" attribute (code 2) is Personal data table (code 31); -First name (code 32) is Richard; -Second name (code 33) is Jones; -Date of birth (code 33) is 01/02/1963; -Social security number (code 34) is 33325333; -Nationality (code 25) is American. The codes (namely 2,31,32,33,34,35) have to be stored sooner or later in ID column. At that time these attributes become entities and are defined by other attributes (eg. the “name” of the entity identified with 82 ID value is Personal insurance ID; the attribute called “name” was defined earlier in ID column see Figure 2 and now it is applied in the attribute column as an entity attribute).

**Figure 5.**
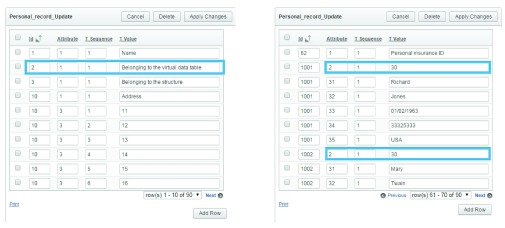
Belonging to the virtual data table.

In the fourth step, the attributes are assigned to each virtual data table using a previously introduced attribute called “belonging to the virtual data table” ( Figure 5).

The following context can be read out: The entities identified with 1001 and 1002 ID values belong to the same virtual data table. With these steps the developer can design one data table to store each entity, attribute and value in a database. Oracle Apex automatically supply each record with row IDs. The above described method can be applied manually. For the automatic conversion (for not primarily cloud-based applications) we created a Java code below ^7^:


public static String getEntityName ( )
throws Exception
{ 
Connection conn = broker.getConnection ( );
PreparedStatementpstmt = 
conn.prepareStatement ("select *from joker"); 
ResultSetrs = pstmt.executeQuery ( );
inti = 0; 
while (rs.next ( )) { 
i++; 
} 
System.out.println ("number of records:" + i); 
broker.freeConnection (conn); 
return ""; 
} 
public static void insert JokerRow 
(Integr GROUP_ID, Integer UNIQ_ID, 
Integer FIELD_ID, Integer ARRAY_INDEX,
String SEEK_VALUE, String FIELD_VALUE)
throws Exception { 
if (GROUP_ID == null) pstmt.setNull (1, 2); 
else pstmt.setInt (1, GROUP_ID.intValue ( ));
if (UNIQ_ID == null) pstmt.setNull (2, 2);
else pstmt.setInt (2, UNIQ_ID.intValue ( )); 
if (FIELD_ID == null) pstmt.setNull (3, 2); 
else pstmt.setInt (3, FIELD_ID.intValue ( ));
if (ARRAY_INDEX == null) pstmt.setNull (4, 2); 
else pstmt.setInt (4, ARRAY_INDEX.intValue ( )); 
if (SEEK_VALUE == null) pstmt.setNull (5, 12); 
else pstmt.setString (5, SEEK_VALUE); 
if (FIELD_VALUE == null) pstmt.setNull (6, 12);
else pstmt.setString
(6, FIELD_VALUE); pstmt.execute ( ); 
} 
public static void readFile ( )
throws Exception 
{ 
File f = new File ("data.txt"); 
BufferedReaderbr = new BufferedReader 
(new FileReader (f)); 
while (br.ready ( )) { 
String line = br.read Line ( ); 
int GROUP_ID = Integer.parseInt
(line.substring (0, 10)); 
int UNIQ_ID = Integer.parseInt 
(line.substring (11, 21)); 
int ARRAY_INDEX = Integer.parseInt 
(line.substring (22, 32)); 
String FIELD_VALUE = line.length ( ) > 32? 
line.substring (33, line.length ( )): " "; 
insertJokerRow (Integer.valueOf (GROUP_ID), 
Integer.valueOf (UNIQ_ID), null, 
Integer.valueOf (ARRAY_INDEX),
null, FIELD_VALUE); 
} 
br.close ( ); 
}


## Results

**Figure 6.**
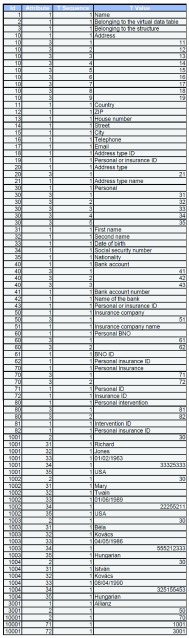
Physical data storage structure.

The resulting table structure is called JT structure ( Figure 6). The result from automatic conversion is a physical data table which uses 6 columns. In cloud, Oracle Apex automatically add row IDs and we introduced "belonging to the virtual data table" attribute instead of Group IDs. In cloud we prefer to use only 4 columns to store each data in a database.

The JT logic-based databases can be defined using primitive relation scheme known as a three-tuple according to Paredaens (1989) ^8^ concept:

*PRS* = ( *ω*, *δ*, *dom*)

where

*ω* is a finite set of attributes, in our case, it is the set of entities from the ATTRIBUTES virtual data table.

*δ* is a finite set of entities, in our case, it is a set of virtual records.

*dom* : *ω* *→* *δ*

is a function that associates each attribute to an entity; it can be interpreted as a predefined set of attributes called "1:N registry hive". This function is used to maintain the entities in the virtual data tables.

A relation scheme (or briefly a relation) is a three-tuple RS=(PRS,M,SC)

where

PRS is a primitive relation scheme; M is the meaning of the relation. This is an informal component of the definition, since it refers to the real world and since we will describe it using a natural language. SC is a set of relation constraints. From the JT physical data table, the following definitions can be read out:

• Virtual record is set of the physical records which have the same ID value.

• Virtual data table is set of the virtual records which have the same value of the "belonging to the virtual data table" attribute.

**Thesis:** In the JT structure, each attribute needs only one index for indexing in the database.

**Figure 7.**
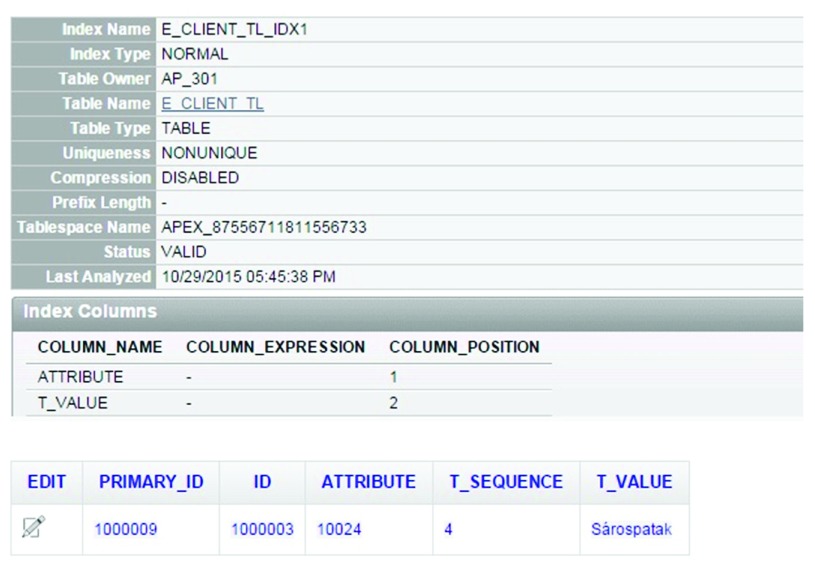
Indexing a record.

**Proof using mathematical induction:** It is obvious the statement is true for the case of one record stored in a data table (according to the RDBMS structure where the developers use more indexes to indexing more attributes). In this case the data table appears as shown in Figure 7.

**Figure 8.**
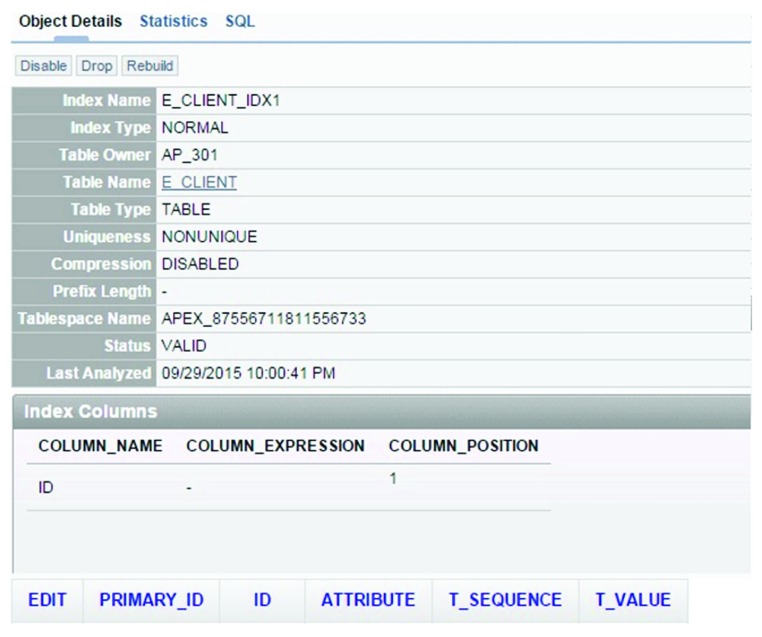
ID usage.

Index= attribute (num) + value (varchar 2) In view of entity, an ID (numerical) index is also used in JT logic-based systems. This ID does not depend (no transitive dependency) on any attribute. Thus, the entities of the virtual data tables meet the criteria of the third normal form ( Figure 8).

**Figure 9.**
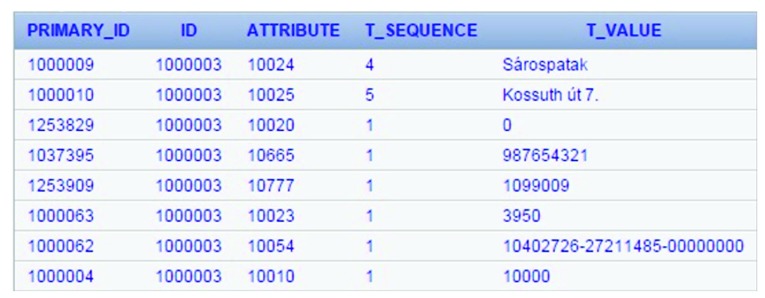
New entity.

**Figure 10.**
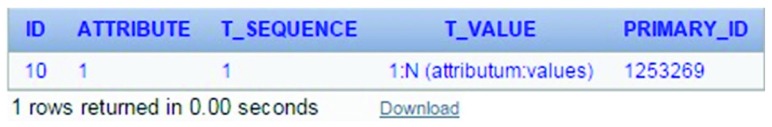
New attribute.

**Figure 11.**
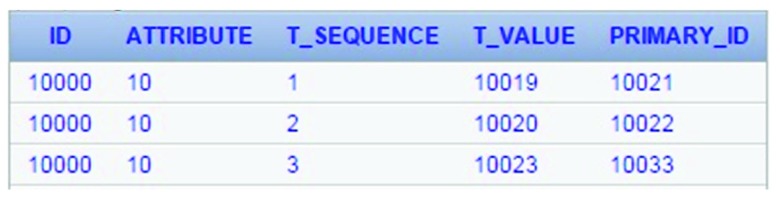
New virtual data table.

The modes of the expansion of a data table are: -input new entity ( Figure 9); -input new attribute ( Figure 10); -input new virtual data table ( Figure 11).

The indexing is correct in case of n+1 record expansion also. With JT logic the user is able to use only one physical data table to define each virtual data table in a database. Therefore, since only one index is required to index each attribute, the statement of the thesis is true in every case of the JT logic-based data table according to the principle of mathematical induction below. Thesis: For n=1 ergo;

                          1 + 2 + .. +
*n* =
*n* * (
*n* + 1)
*/*2

substituting one into the equation we get:

                                    1 = 1 * (1 + 1)
*/*2

result of the operation is 1=1, that is, the induction base is true.

Using proof by induction we can now show that this is true for the following equation:

n = k where k is a optional but fixed natural number. Therefore, we know that the following operation is true:

                          1 + 2 + .. +
*k* =
*k* * (
*k* + 1)
*/*2

Finally using n=k+1 we can prove our assumption to be true:

                  1 + 2 + .. +
*k* + (
*k* + 1) = (
*k* + 1) * (
*k* + 2)
*/*2

The above induction proof shows:

                  1 + 2 + .. +
*k* + (
*k* + 1) =
*k* * (
*k* + 1)
*/*2 + (
*k* + 1)

Conducting the mathematical operations we obtain the following:

                  1 + 2 + ..
*k* + (
*k* + 1) = (
*k* * ((
*k* + 1)
*/*2) + 2 * (
*k* + 1))
*/*2 =

                        (
*k* *
*k* +
*k* + 2
*k* + 2)
*/*2 = (
*k* *
*k* + 3
*k* + 3)
*/*2

Conducting the mathematical operations on the other side we obtain the same:

                       (
*k*+1)*(
*k*+2)
*/*2 = (
k*k+2
*k*+
*k*+2)
*/*2 = (
*k*+
*k*+3
*k*+2)
*/*2

Thus, the induction step is true. Given that both the induction base and the induction step are true, the original statement is therefore true. In the present study, we explained the JT data storage logic. In our other study we focused on the query tests. Our previous results 7 show that from 18000 records the relational model generates slow (more than 1 second) queries in Oracle Apex cloudbased environment while JT logic based databases can remain with the one second time frame.

## Discussion and conclusions

Using the developed database management logic, each attribute needs only one index for indexing in the database. JT allows any data whether entity, attribute, data connection or formula, to be stored and managed even under one physical data table. In the JT logic based databases, the entity and the attribute are used interchangeably, so users can expand the database with new attributes after or during the development process. With JT logic, one physical data storage is ensured in SQL database systems for the storage and management of long term scientific information.

## Data Availability

The data referenced by this article are under copyright with the following copyright statement: Copyright: © 2016 Mátyás B et al.

Data associated with the article are available under the terms of the Creative Commons Zero "No rights reserved" data waiver (CC0 1.0 Public domain dedication). 

*Figshare*: A novel data storage logic in the cloud. doi: 10.6084/m9.figshare.3119086 ^9^
