# Supplementary material for: A novel data storage logic in the cloud
Source: F1000Res. 2016 Mar 29;5:93. Originally published 2016 Jan 21. [Version 2] doi: 10.12688/f1000research.7727.2 (PMC5583739; doi:10.12688/f1000research.7727.2)
Supplement: Peer review history

# Peer review: Referee response for version 2

**DOI:** 10.5256/f1000research.8995.r13081

**Published:** 2016-04-06

**Authors:** Jan Lindström

**Affiliations:** 1 MariaDB Corporation, Espoo, Finland

In my first review I requested full and significant rewrite of the paper. This has not happened. Authors did add some new material and video, both useless for validating the correctness and usefulness of the proposed method.

Firstly, research question is missing, what are the problems the proposed method tries to

solve?

Secondly, the transformation of the traditional relational model to proposed model is not described clearly enough. It seems that there are some rules how relations and their attributes are stored to new structure, but this is not described clearly enough. Paper should list clearly set of rules that are used and give examples how these rules are applied.

Third, usefulness of  the proposed method is not clear. Sure you can have only one index, but how you do simple queries like select first_name,street from Personal p, Address a where a.id = p.id is executed? How user could know what ID some attribute now has? How the created one index can be used to perform simple primary key or foreign key queries. How constraints are enforced ?

Finally, what are the use cases for JT logic and how the proposed method improves the state-of-the-art i.e. compared to relational model or object oriented model? This question remain fully open based on this paper.

This paper does not successfully fulfill requirements of the scientific paper. At its current form, this looks more like a marketing material.

I have read this submission. I believe that I have an appropriate level of expertise to state that I do not consider it to be of an acceptable scientific standard, for reasons outlined above.

# Peer review: Referee response for version 1

**DOI:** 10.5256/f1000research.8321.r12373

**Published:** 2016-02-25

**Authors:** Kavita Sunil Oza

**Affiliations:** 1 Department of Computer Science, Shivaji University, Kolhapur, Maharashtra, India

Work demonstrated in the paper is good and well explained. Complexity of work is not mentioned (algorithmic complexity) but this is not necessary as we already have high speed processors and time complexity may not matter much *. *Some more references should have been added but not mandatory as number of references are sufficient.

I have read this submission. I believe that I have an appropriate level of expertise to confirm that it is of an acceptable scientific standard.

# Peer review: Referee response for version 1

**DOI:** 10.5256/f1000research.8321.r12375

**Published:** 2016-02-15

**Authors:** Jan Lindström

**Affiliations:** 1 MariaDB Corporation, Espoo, Finland

In this paper authors introduce a new logic called Joker Tao (JT) which provides universal data storage for cloud-based databases. However, the paper is very poorly written. Firstly, the proposed logic is not presented detailed enough for the reader to understand and validate the method. Authors should research how relational model is presented and based on rigorous relational calculus and algebra. Based on this research, this paper should be rewritten based on rigorous mathematical foundation and give clear examples. Secondly, one table based example is far from convincing and provided Java-program is unnecessary. Length of the paper should be greatly increased to contain detailed description of JT method and give examples. Lastly, presentation is so poor that is not even clear how queries to resulting JT structure can be executed. To be honest, currently paper looks more like computer generated rubbish than a real scientific paper.

I have read this submission. I believe that I have an appropriate level of expertise to state that I do not consider it to be of an acceptable scientific standard, for reasons outlined above.
